# Exploring juventology: unlocking the secrets of youthspan and longevity programs

**DOI:** 10.3389/fragi.2024.1379289

**Published:** 2024-04-04

**Authors:** Sebastian Brandhorst, Valter D. Longo

**Affiliations:** Leonard Davis School of Gerontology, Longevity Institute, University of Southern California, Los Angeles, CA, United States

**Keywords:** aging, longevity, gerontology, juventology, healthspan

## Abstract

In recent decades, the study of biological aging has evolved from simplistic theories like the free radical theory to more complex and nuanced perspectives. In particular, the identification of evolutionary conserved genes and signaling pathways that can modulate both lifespan but also healthspan has resulted in the expanding understanding of the link between nutrients, signal transduction proteins, and aging along with substantial support for the existence of multiple “longevity programs,” which are activated based on the availability of nutrients. Periodic fasting and other dietary restrictions can promote entry into a longevity program characterized by cellular protection and optimized function, and the activation of regenerative processes that lead to rejuvenation. This review discusses the idea of juventology, a novel field proposing the existence of longevity programs that can maintain organisms in a highly functional state for extended periods of time. Drawing upon research on *Saccharomyces cerevisiae* and other model organisms, the review explores the distinctiveness of juventology from traditional aging-centered views. The focus on the “age of youth” challenges conventional thinking and opens new avenues for understanding and extending the period of peak functionality in organisms. Thus, a “juventology”‐based strategy can complement the traditional gerontology approach by focusing not on aging but on the longevity program affecting the life history period in which mortality is very low and organisms remain youthful, healthy, and fully functional.

## 1 Introduction

Aging is generally considered a time-related, non-adaptive, and deteriorating process resulting from the decline in the force of natural selection. *Theories of Aging* try to explain the various aspects of the aging-related gradual deterioration of functional characteristics in living organisms (“senescence”). Because the process of senescence is complex, derives from a variety of different mechanisms and may exist for a variety of different reasons, the most proximal estimation of the etiology of aging is by accepting that all aging-theories contribute to a holistic understanding of the aging process. Existing theories of aging can be generalized into the programmed and stochastic theories of aging and are classified into *evolutionary*, *physiological, structural* and *functional* changes. Processes that emphasize the role of intrinsic timing mechanisms and signals (for example, through hormone signaling), accidental chance events, programmed genetic signals, nuclear or mitochondrial DNA mutations or damage, the accumulation of damaged and abnormal proteins, cross-linkage, glycation, waste accumulation, general molecular wear and tear, free radical formation, and specific cellular components such as genes, chromosomes, mitochondria, or telomeres are all included into these aging theories at the cellular level. Physiologic processes that may explain aging include oxidative stress, immunology, neuro-endocrinology, the metabolism, and insulin signaling pathways. Cellular senescence, one aspect of organismal senescence, is characterized by the arrest of cellular proliferation in response to endogenous but also exogenous stress incl. including telomere dysfunction, oncogene activation and DNA damage. Cellular senescence is understood as a highly regulated program that includes the activation/deactivation of signaling cascades that modulate gene expression profiles, transcription factor activities, or extrinsic activities, most prominent the activation of the senescence-associated secretory phenotype (SASP) which can amplify cell cycle arrest; thus cellular senescence is one of the factors that contributes to organismal sentence by impairing tissue regeneration and the occurrence of chronic age-associated diseases. Cellular theories of aging consider the increasing proportion of cells that reach senescence the dominant driver of age-related decline in physical function.

## 2 Classic theories of aging

Evolutionary aging theories try to explain the differences in aging rates and longevity across different biological species (e.g., mice and humans) through the interplay of mutation- and selection-processes. For example, observations of the “suicidal” life cycles of species like the pacific salmon have promoted the idea that sexual reproduction may come with a cost for species longevity. Thus, in addition to mutation and selection, the reproductive cost, or, more generally, the trade-offs between different traits of organisms may also contribute to the evolution of species aging and longevity (Gavrilov and Gavrilova, 2002). The “disposable soma theory” was proposed by Kirkwood in 1977 and describes that all living organisms have limited energy (Kirkwood, 1977) which must be balanced between maintenance and reproduction, thus allowing reproduction when nutrients are sufficiently provided; the idea here is to secure survival of the offspring within a nutrient-rich environment. Vice versa, in conditions with reduced nutrient availability, the reproductive rate is then reduced to instead maximize cellular function. It is the compromise in allocating energy between reproduction and repair that causes the body gradually to deteriorate with age.

The logical foundations for most of the modern evolutionary theories of aging were completed relatively late in the 1950s and almost a century after Darwin suggested his theory of biological evolution (Darwin, 2003). The biological evolution of aging was initially studied in a purely theoretical and non-experimental way by Weismann (Programmed death theory, 1882; later adapted), Medawar (Mutation accumulation theory, 1952), Williams (Antagonistic Pleiotropy theory, 1957) and others. A detailed overview about evolutionary aging theories is provided by Gavrilov et al. (Gavrilov and Gavrilova, 2002). Evolutionary theories of aging were subsequently (although only partially) tested using the fruitfly *Drosophila melanogaster* (Stearns et al., 2000) and on natural populations of *Poecilia reticulata* guppies (Reznick et al., 2001). Selection for later reproduction (artificial selection of late-born progeny) produced, as expected, longer-lived fruit flies while placing guppies in a more dangerous environment with high extrinsic mortality redirected evolution to a shorter lifespan in the following generations. These experiments found that aging and lifespan do evolve in subsequent generations of biological species in a theoretically predicted direction depending on the particular living conditions. The Antagonistic Pleiotropy theory views aging as the end result of genetic trade-offs between genes/alleles that benefit fertility/reproduction early in life at the cost of physical deterioration of the body later in life; evolution thus prioritizing the continuation of a species/community over long-term survival of the individual (Mitteldorf, 2019). One prominent critique of this theory is that it is inconsistent with observations that some of the mutations that extend longevity can so without do causing growth or fertility disadvantages. Recently, Mitteldorf has proposed an alternative hypothesis for the Antagonistic Pleiotropy theory that proposes that “*Antagonistic pleiotropy evolves as an evolvability adaptation that protects long term group-level benefits from being lost to short term individual selection*” (Mitteldorf, 2019).

The evolutionary theories of aging are closely related to the genetics of aging because biological evolution can only be possible through inheritable manifestations of aging. Programmed aging theories propose that senescence is the final destination in a developmental pathway that culminates in death. These theories suggest that aging is under control of “biological clocks” which operate throughout the lifespan of an organism. The regulatory effects of these clocks seem to be depending on changes in the gene expression pattern of systems involved in cellular-maintenance, -repair, and -defense. One such theory is the “Reproductive-Cell Cycle Theory” which argues that cell growth, development, and death are under the regulation of reproductive hormones derived of the hypothalamic-pituitary-gonadal axis. According to this theory, reproductive hormones, such as estrogens, progestagens, androgens and gonadotropins, and their receptors, have an essential function in promoting growth and development of the organism; normal function in early life is important in order to achieve a maximal reproductive rate. With the onset of hormonal changes (in men around age 30 and in women when they reach menopause, around age 50), the hypothalamic-pituitary-gonadal axis becomes unbalanced, cellular growth and development become dysregulated, cellular-dysfunction and cell-death are increasingly more prominent and thus promote senescence (Atwood and Bowen, 2011). Supporting evidence for the reproductive-cell cycle theory includes studies that demonstrate that women with menopause occurring later in their life show less heart disease and have less stroke incidences, are less prone to dementia, and experience less osteoporosis. Conversely, early surgical menopause has been demonstrated to increase the incidence of these diseases (Ossewaarde et al., 2005). In addition, studies in support of the theory have shown that suppressing growth hormone, regulated by the neuro-secretory nuclei of the hypothalamus, and insulin-like growth factor 1 (IGF-I) signaling (a part of the growth hormone axis), such as caused by caloric restriction or exercise, increases lifespan (Atwood and Bowen, 2011). Another programmed theory of aging is related to such a “fixed” life span model. The developmental–genetic theory of aging proposes that the genetically programmed induction of senescence occurs during adult lifespan, which results in either the activation, or suppression, of specific “aging” genes. Support for this theory comes from studies that indicate that longevity in humans seems to be hereditable related to the presence of specific genes. One such “aging gene” is the human leucocyte antigen (HLA), which showed significantly high levels in a study of old (over the age of 85) healthy subjects (Ricci et al., 1998). However, research showing that physical fitness improves longevity in humans goes against this theory (Reimers et al., 2012). In addition, programmed aging theories and evolutionary theories do not exclude each other but can instead display additive features; for example, women with menopause occurring later in life have a lower incidence of age-related diseases but also frequently have single nucleotide polymorphisms (SNPs) in repair genes (Louwers and Visser, 2021).

Stochastic theories position environmental impacts, which induce cumulative damage at various levels throughout the entire organism, as the cause of aging; examples of which include oxygen radicals (widely known as free radicals and countered by the even more prominent antioxidants) that can damage DNA (for example, via cross-linking), cells, and tissues. The “Chromosomal Alterations Theory” proposes that normal aging is directly related to alterations in the chromosomal structure: deletions, mutations, translocations, and polyploidy acquired chromosomal instabilities contribute to gene silencing or gene specific expression over time (Cefalu, 2011). Yet, at the population level, one can observe that mortality rates in the very elderly actually decline (Partridge et al., 2005), which contradicts the assumption that the accumulation of damage itself, or life-shortening mutations, regulates lifespan. These stochastic theories likely also hold true when viewed from a physiological perspective as metabolic processes change with age and thus can induce additional stochastic accumulation of macromolecular damage. Another popular theory of aging is the autoimmune theory, which is based on the hypothesis that the human body produces auto-antibodies against its own tissues and/or that deficits, primarily in T-cell function, predispose the elderly to the development of infections, chronic disease, cancer, and autoimmune diseases such as rheumatoid arthritis (Kent, 1977).

One of the most common theories of aging is the “Free Radical Theory of Aging”, first proposed by Harman in the 1950s (Harman, 1956). The theory states that highly reactive oxygen-derived substances (free radicals) result in the accumulation of protein, lipid, and DNA damage as a result of normal cellular metabolism. In later years, the theory was extended to include the role of mitochondrial free radical production (Harman, 1972). Free radicals are mainly produced inside the cell organelles, in particular inside the mitochondria, which convert energy for the cell into adenosine triphosphate (ATP). Oxidative phosphorylation, the process in which ATP is produced, involves the shuttled transport of protons (hydrogen ions) through the electron transport chain across the inner mitochondrial membrane. For ATP production, electrons are passed through a series of proteins; each acceptor protein along the chain has a greater reduction potential than the previous one. The last destination for an electron along the electron transport chain is an oxygen molecule which is then reduced to produce H_2_O. The reduction of oxygen and the transported electrons however is not 100% efficient and, in about 0.1%–2%, oxygen is instead prematurely and incompletely reduced to produce the superoxide radical (·O2-), mostly documented for Complex I and Complex III (Gomez and Hagen, 2012). Reactive oxygen species are therefore a byproduct of normal cellular metabolism. Superoxide is not particularly reactive by itself, but plays a role in hydrogen peroxide formation (H_2_O_2_), which can leak from the mitochondria into the cell. It is postulated that reactive oxygen may be a signal for aging and its levels in tissues may determine the aging process and lifespan.

However, multiple experiments critically question the free radical theory. First, the over-expression of major antioxidant enzymes (e.g., superoxide dismutase or catalase), does not extend the lifespan of mice (Perez et al., 2009) and mitochondrial superoxide dismutase (Sod-2) haplo-insufficiency does not accelerate murine aging, even in mice with dysfunctional telomeres (Guachalla et al., 2009). Even more surprisingly was that the deletion of Sod-2 extended the lifespan in the nematode *Caenorhabditis elegans* (Van Raamsdonk and Hekimi, 2009). It was also reported that RNAi of five genes encoding components of mitochondrial respiratory complexes I, III, IV, and V leads to increased life span in flies. Long-lived flies with reduced expression of electron transport chain genes do not consistently show a reduced assembly of respiratory complexes or reduced ATP levels. In addition, extended longevity is not consistently correlated with increased resistance to the free-radical generator paraquat (Copeland et al., 2009). These results are in agreement with previous papers showing that antioxidant over-expression causes minor effects in life span extension in yeast, flies, and mice compared to those caused by mutations in signal transduction genes. For example, yeast and flies that overexpress superoxide dismutases and catalases, which scavenge free radicals, survive only approximately 5%–30% longer than wild-type flies (Sun and Tower, 1999; Sun et al., 2002; Fabrizio et al., 2003), whereas mutations in central nutrient-response pathways cause a twofold to threefold lifespan extension (Fabrizio et al., 2001; Tatar et al., 2014). It is likely that the increased protection against superoxide must be accompanied by a number of other changes to be effective in life span extension.

Theories of biological aging, including the free radical theory of aging, the disposable soma, and antagonistic pleiotropy theories, have been developed to provide conceptual frameworks to explain the aging processes that are consistent with the acceleration of damage over time and dysfunction as the force of natural selection declines. Yet, what if we would consider biological aging to be the ending (or at least a partial inactivation below its functional threshold) of a “longevity program” whose scope is to maintain the organism functionally young/youthful (Longo et al., 2005)? In this context, the term “program” should be considered as an evolutionary conserved framework that regulates the interaction between external factors (such as macronutrient availability) and the activation/deactivation of cellular signaling cascades that can modulate protein expression profiles and longevity (Longo and Finch, 2003). Such a program could allow organisms to prolong their normal lifespan by entering into a “maintenance mode” that is associated with changes such as hypometabolism, high stress-resistance significantly reduced fertility, as well as increased somatic maintenance. Mutations in genes previously identified to extend lifespan could then affect this program so that individuals enter this maintenance mode irrespective of the existing environmental conditions (Longo and Finch, 2003; Kenyon, 2005). This idea of a programmed longevity theory is consistent with a role for free radicals in causing age-dependent damage, but further proposes that the primary cause of aging is the genetically programmed inactivation or decline in a system over time that normally prevents damage by regulating protection, repair and replacement of genes. Notably, this theory is supported by several studies that are consistent with the existence of programmed longevity. When fruit flies are calorie-restricted (a dietary intervention that is known to extend lifespan) for 48 h at two different ages, their mortality is indistinguishable from that of flies that are calorie-restricted for their entire lifespan (Mair et al., 2003; Mair et al., 2005); and similar results are observed for the chronological lifespan of calorie-restricted yeast (Leonov et al., 2017; Arlia-Ciommo et al., 2018). It is possible that the reversed mortality rates of *D. melanogaster* that have been switched to calorie restriction are a consequence of a reversal of the damage (Mair et al., 2003; Partridge et al., 2005). However, this ability to reverse the damage accumulated since birth so rapidly would further indicate that organisms only maintain sub-optimal level of, and/or minimally active repair and replacement systems. In rodents, late-onset calorie restriction (CR) may extend health and lifespan (although largely depending on the age of CR initiation) (Ingram and de Cabo, 2017), whereas the overexpression of antioxidant enzymes causes little or no lifespan extension in mice (Gallagher et al., 2000; Perez et al., 2009). If free radicals were the primary cause of aging we would expect the effect of antioxidant enzymes on lifespan to be closer to that of mutations in signal-transduction genes. Therefore, the data are consistent with the existence of programs that optimize the “normal” lifespan and that these programs can respond to starvation conditions by extending longevity through the regulation of many repair and protection systems which include, but not exclusively, antioxidant enzymes (Austad, 2004; Bredesen, 2004). The identification of the longevity program might be particularly important for the identification of the primary regulators of aging and age-related diseases.

Although the existence a longevity program is largely consistent with the natural selection theory and thus may appear to be just another way to explain aging, notable differences include the reduced emphasis on cellular/organismal senescence but instead focuses more on protection, repair, and replacement events that help to keep organisms biologically young. This idea was proposed in 2019 and appropriately termed “*juventology*” (a.k.a. “the study of youth”) as derived from the Latin *iuventus* or “the age of youth.”

## 3 Theories of aging vs. juventology

### 3.1 Programmed longevity and “juventology”

The paradigm of longevity programs opens up new vistas for understanding interventions that extend lifespan without instigating adverse effects. While traditional aging research has often fixated on combating free radicals and oxidative stress, juventology suggests that the most effective pro-longevity interventions induce alternate survival phases. The exploration of longevity programs in model organisms reveals a complex network of cellular responses and adaptive strategies that challenge the somewhat conventional theories of aging. Especially, the interplay between nutrient availability and the activation of specific longevity programs is not just a passive response but instead highlights a sophisticated network of cellular events that over the course of the lifespan can result in a healthier aging phenotype and increased longevity. In *E. coli*, *Saccharomyces cerevisiae*, and *C. elegans*, starvation, the most severe form of dietary restriction, causes a major lifespan extension (Longo and Mattson, 2014; Longo and Panda, 2016).

The single-celled eukaryote *S. cerevisiae* has served as an invaluable model organism to unravel some of the mysteries of longevity programs. The work by Longo, Mitteldorf, and Skulachev in 2005 demonstrated that the choice of nutrients in the growth medium significantly influences the organism’s lifespan (Longo et al., 2005): in a glucose-rich environment, *S. cerevisiae* adopts a relatively low protection mode, surviving for approximately 6 days. However, a remarkable shift occurs when the organism is exposed to a water medium: yeast cells not only exhibit a substantial increase in stress resistance but also experiences an extension in lifespan. Moreover, the period in which cells can reproduce and form colonies is significantly prolonged. This intriguing duality in longevity programs challenges the conventional aging-centered perspective by introducing the concept that the trajectory of an organism’s aging is not predetermined but can be influenced by environmental cues. Subsequent research has elucidated that some of the pro-longevity effects of starvation result from deficiencies in amino acid availability (particularly serine, threonine, and valine but also methionine) which effect Tor‐S6 kinase and other intracellular nutrient signaling pathways (Mirisola et al., 2014; Ables and Johnson, 2017; Green et al., 2022). Additionally, the reduction in glucose levels and the consequent down‐regulation of Ras‐PKA signaling are key changes that play a substantial mediating role in the fasting‐dependent effects on yeast aging (Longo and Mattson, 2014). While in mammals nutrient signaling is much more complex than in yeast (Fontana et al., 2010), the fundamental role of macronutrient availability on organismal/cellular aging may be evolutionary conserved across species. As such, deficiencies in methionine and other amino acids can reduce growth hormone (GH) signaling and consequently the levels of the insulin‐like growth factor 1 (IGF‐1), thereby in turn regulating the activity of the mTor‐S6 Kinase pathway; with effects analog to what has been shown in yeast. These evolutionary conserved signaling cascades thus regulate longevity across multiple species, and therefore not surprisingly, protein or amino acid restriction also extends longevity in flies and mice and recent epidemiological studies indicate that it can reduce the risk of cancer and overall mortality in humans (Miller et al., 2005; Grandison et al., 2009; Levine et al., 2014; Song et al., 2016).

Importantly, the evolutionary conserved effects of protein/amino acids restriction on longevity are consistent with the reduced morbidity phenotype observed in both mice and humans with defects in the pathways they activate. One of the most prominent pathways includes growth hormone receptor (GHRD) signaling, whose inactivation causes protection from both cancer and diabetes (Guevara-Aguirre et al., 2011; Bartke et al., 2013). Mice with GH or GHR deficiency also display an over 40% longevity extension, which has not yet been determined for human subjects with GHRDs (Bartke et al., 2002; Bartke, 2008; Guevara-Aguirre et al., 2011; Arum et al., 2014). Because mammalian GHRD model organisms are born with this mutation, it is possible that a significant portion of the longevity phenotype is due to developmental effects of the mutation, particularly considering that methionine restriction early in life can result in lifespan extension. However, rapamycin, calorie restriction, or periodic cycles of a Fasting Mimicking Diet (FMD) started at middle age can extend both longevity and healthspan probably at least in part by inhibiting GH‐IGF‐1 and/or mTor‐S6K signaling (Harrison et al., 2009; Miller et al., 2011; Colman et al., 2014; de Cabo et al., 2014; Brandhorst et al., 2015; Mattison et al., 2017), strongly suggesting that the involvement of these pro‐growth genes in the acceleration of the aging process can be independent of developmental effects.

Juventology is fundamentally different from “aging‐centered” theories of aging for two reasons: (1) alternative lifespan programs, such as those entered in response to starvation, can be independent (or are at least partially independent) of aging itself. As an example, one could visualize the use of target-specific pharmaceuticals or systemically broader acting periodic fasting intervals modulate the mTor‐S6K and PKA pathways (Ros and Carrascosa, 2020), which in turn can promote regeneration and rejuvenation. Notably, this can be achieved even in an organism with a high rate of aging. Thus, even in an accelerated aging phenotype, a longer healthspan and lifespan may be accomplished by periodically activating regenerative and rejuvenating processes. (2) Juventology shifts the focus from an “old or older age” paradigm characterized by high degrees of dysfunction and subsequent high morbidity and mortality, instead to the period in life during which both morbidity and mortality are very low and only difficult to detect. Diseases in humans are generally rare before the (biological) age 40, but comorbidities are common after age 65, yet no specific field of science is focusing on how evolution resulted in a program that is extremely efficient in preventing disease for the first 40 years of life and how that program may be modulated and extended by dietary, pharmacological, or other interventions. On the one hand, developmental biology focuses on the biological process from embryo to (young) adult stage and generally does not include this important field. On the other hand, biogerontology the biological basis of aging and age-related diseases. Thus, juventology presents a complementary field to both gerontology and developmental biology that focuses on the period of organismal life when the force of natural selection is high and body functions remain maximized.

This programmed longevity and juventology understanding offers approaches that may extend lifespan without promoting adverse effects. Historically, significant efforts in biogerontolgy have focused on the damaging effects of free radicals and other oxygen species along with a quest for antioxidant enzymes and drugs that can counter these effects. Although free radicals are without a doubt among the most potent pro‐aging molecules, treating model organisms with rapamycin, spermidine, metformin, and NAD‐related molecules, or dietary restriction, has been proven to be more effective than antioxidants in extending healthspan. Instead of simply targeting specific toxic molecules [which besides being promotors of biological aging may also have a beneficial role such as killing bacteria or malignant cells; i.e., reactive oxygen species modulate several aspects of T cell-mediated immunity (Yarosz and Chang, 2018)], most successful pro‐longevity interventions initiate highly coordinated signaling cascades that can enhance cellular resistance, protection, and repair evolved to respond to starvation conditions or other insults (Miller et al., 2005; Colman et al., 2009; Harrison et al., 2009; Miller et al., 2011; Cheng et al., 2014; Colman et al., 2014; Longo and Mattson, 2014; Brandhorst et al., 2015; Mattison et al., 2017; Madeo et al., 2018). Autophagy as well as the tissue-specific induction of stem cell-based regeneration have been established as key outcomes for restoring an organism to a younger, maybe even rejuvenated state, following the induction of these programs (Wang et al., 2019). Intricate and finely tuned cellular response networks, which maintain the capacity to be modulated by healthspan-extending interventions, challenge more reductionist approaches that only target specific pro-aging molecules. Instead, these prolongevity pathways engage a holistic approach, activating systemic and cell type-specific programs that respond to conditions of starvation or other stressors, underscoring the importance of cellular adaptability in the face of changing environmental cues. The ability to shift gears and enter alternate phases of survival, cellular resilience, and rejuvenation highlights a currently largely untapped therapeutic potential within our cells.

### 3.2 Juventology strategies to improve human aging

The juventology strategy could eventually play a complementing role in medicine that combines the selective targeting of specific enzymes or processes that drive disease (traditional medicine approach) with the induction of programs that allow organisms to remain highly functional, resilient, and adaptable (juventology). Although this program allows *S. cerevisiae* to live for 6 days but remain highly protected for 3 days, and mice to live 2.5 years while remaining highly protected and functional for about 1 year, evidence whether or not the same program extends the human youthspan period remains largely unclear. Although a major extension of youthspan may sound unrealistic, it has already been achieved in mice, monkeys and possibly humans treated with dietary restrictions, periodic fasting, pro‐longevity drugs, or bearing specific mutations. Most notably, recent studies in humans show that dietary restriction or a fasting-mimicking diet applied for just 3 to 5 days-lasting cycles can reduce biological aging (Waziry et al., 2023) (and NCOMMS-21-10638C, *accepted for publication, citation coming*). However, because most studies focus exclusively on lifespan and healthspan, the effects of most prolongevity interventions on youthspan remain elusive and methods to study youthspan outcomes are unspecified. As previously proposed (Longo, 2019): using simple model organisms, such as *C. elegans*, *D. melanogaster*, or *S. cerevisiae* in which large populations can be studied, youthspan could be measured based on age‐specific mortality rates combined with more specific measurements that assess macromolecular damage, stress resistance, cognition, and reproductive function. In more complex model organisms, including mice and other mammals, youthspan could be measured using similar metrics for stress resistance, mortality, fertility, and macromolecular damage, but these measurements require additional assays, such as running speed and other physical tests, to establish a meaningful database that correlates outcomes to youthspan. These assays can, or maybe even should, partially overlap with tests already used to assess frailty progression in the elderly (Dolenc and Rotar-Pavlic, 2019), although modifications to existing protocols need to be made to assess whether the measured function is within the thresholds that can be expected in a young or relatively young person. Examples of such modifications include that walking speed could be replaced with running speed instead, and that strength tests assessing different muscle groups are utilized vs. grip strength alone.

## 4 Discussion

The seminal “hallmarks of aging” article and subsequent follow-up publications (Lopez-Otin et al., 2013; Lopez-Otin et al., 2023) define aging as a process characterized by the occurrence of key features fulfilling the following three premises: (1) an age-associated manifestation, (2) aging can be accelerated via experimental manipulation, and (3) that therapeutic interventions can decelerate, stop, or reverse aging. The recently expanded (from nine to currently twelve) hallmarks of aging include genomic instability, telomere attrition, epigenetic alterations, loss of proteostasis, disabled macroautophagy, deregulated nutrient-sensing, mitochondrial dysfunction, cellular senescence, stem cell exhaustion, altered intercellular communication, chronic inflammation, and dysbiosis (Lopez-Otin et al., 2023). Notably, these hallmarks interact and are not independent from “the hallmarks of health”, which include spatial compartmentalization, maintenance of homeostasis, and adequate responses to stress that reflect a series of dynamic features to maintain an organism-wide equilibrium that precedes disease (Lopez-Otin and Kroemer, 2021). Thus, one could understand the here proposed “juventology”‐based approach that focuses on the longevity program affecting the life history period in which mortality is very low and organisms remain youthful, healthy, and fully functional as a component of these “hallmarks of health”. An extension in youthful lifespan should thus theoretically delay the occurrence of hallmarks of aging within model organisms and humans. One could argue that people living in “Blue Zones”, geographic areas with lower incidences of chronic diseases and an extended life expectancy than in the average population surrounding them, benefit from lifestyle modifications (incl. diet, moderate exercise, social connection, etc.) that activate juventology programs throughout their adolescence, but also provide health benefits in combination with select traits in the very old (Poulain et al., 2021).

**FIGURE 1:**
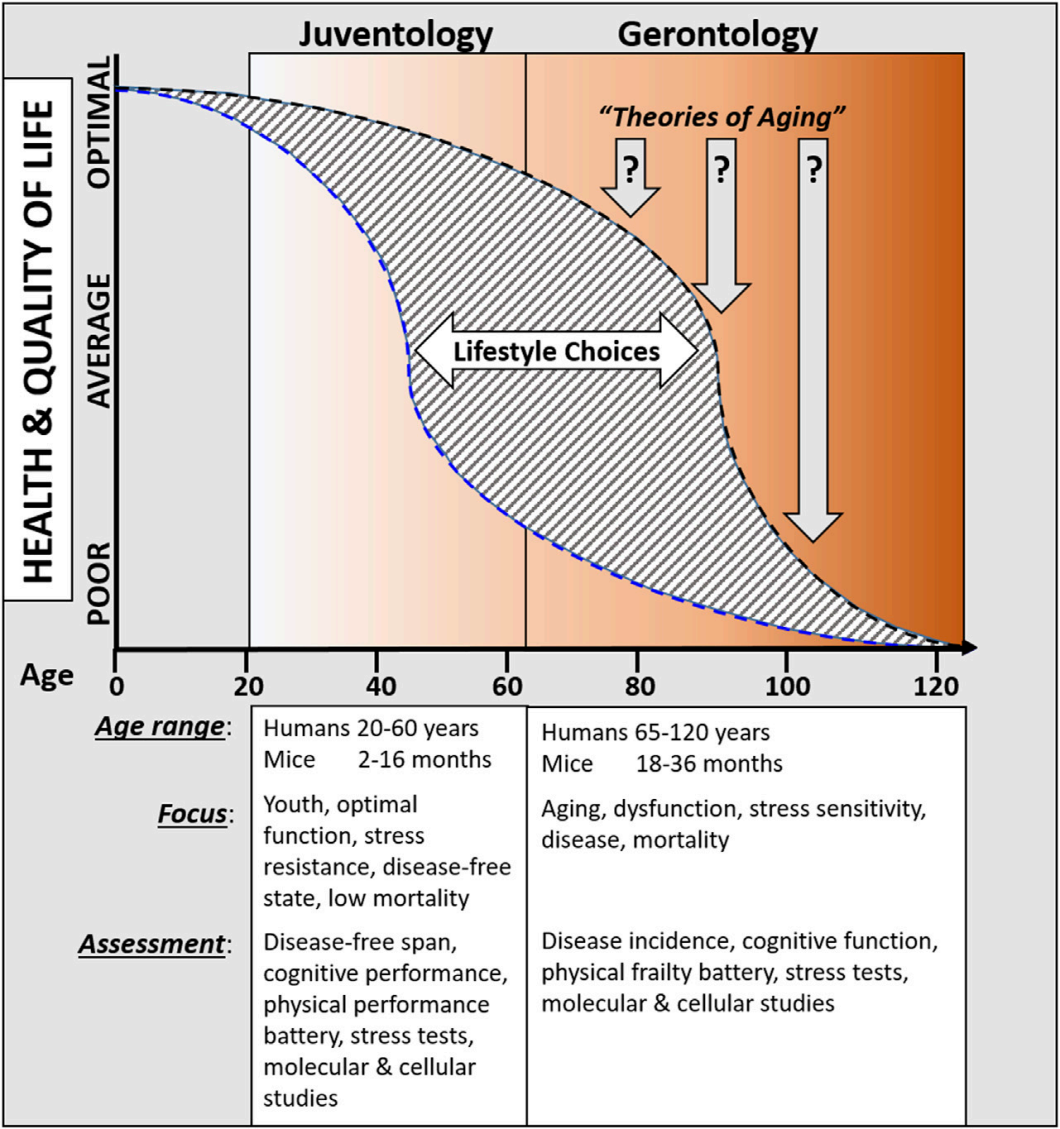
Overview comparing proposed juventology and gerontology approaches.

Periodic fasting and calorie restriction, that go in hand with the decrease in the availability of both proteins/amino acids and glucose levels, promote cells to enter into a stress resistance state which is characterized by the activation of cell protection, regeneration, and rejuvenation processes. Across multiple species, these protective and regenerative mechanisms are activated in part by the down‐regulation of GH, IGF‐1, mTor‐S6K, and PKA signaling cascades, which in turn induces the extension of healthspan. Because these states have evolved to withstand periods of extreme nutrient starvation, they can be viewed as alternative longevity programs activated to maintain cellular “youthspan” until resources that promote proliferative processes become available again. Here, we propose that these a juventology‐based approaches provide complementary strategies to the classic biogerontology approaches to focus on the earlier (i.e., biologically younger) functional period while also studying the later progressively dysfunctional processes that affect health and longevity (Figure 1).

Considering the declining mortality rates in the very old, one could also argue that a reduced central feeding drive with age would increase the relative degree of caloric restriction, thereby promoting a cellular maintenance mode, and leading to enhanced cellular resilience with age. However, it is much more likely that the decrease in appetite as the consequence of poor dental conditions, age-associated changes in taste and smell that influence food choice including the type and quantity of food eaten, as well as medical conditions such as gastrointestinal disease and prescribed medication instead promote malabsorption, anorexia, micronutrient deficiencies, and increased energy and protein requirements (Donini et al., 2003; Levine et al., 2014). Therefore, it is unlikely that a reduced appetite during aging is correlated to the declining mortality rates in the very old.

Important directions for the novel approach to understand senescence could include the addition of in‐depth studies of some of the most remarkable examples of alternate longevity programs in model organisms. Examples of which include the dramatic increases in lifespan such as the spore and dauer phases in starved yeast and worms (Longo, 2003), respectively, that entered by the very long‐lived queen bees (Jemielity et al., 2005), or the dormant diapause of killifish (Hu and Brunet, 2018). In higher eukaryotes, it will also be of great interest to understand how hibernation and the exit from hibernation may affect youthspan, longevity, and biological age (Al-Attar and Storey, 2020): some hibernating mammalian species live up to 9.8-times higher than their expected average lifespan, and a common trait amongst these hibernators species is the depression of their metabolic rate and to initiate cytoprotective responses that maximize survival. The understanding of these processes is likely to have fundamental impact on how we understand organismal development, health, and longevity with the ultimate goal to identify clinical applications to extend our biologically youthful healthspan. The positive impact on individual quality of life, or even the entire healthcare system, that this approach promises are not hard to imagine.

Ultimately, understanding what drives senescence, whether as an individual experience or as population dynamic events, helps us to explore the limit to human longevity: is our species lifespan fixed and will not increase indefinitely or does the deceleration of age-specific mortality in the very old suggest that there is no such limit? Advances in machine learning may eventually provide a better answer to this question but current modeling strategies still largely rely on biased/inaccurate data that limit this approach.

Juventology is different from “agingandhyphen;centered” biogerontology studies and thus also distinct from the classic theories of aging. Juventology focuses on the period in life during which both morbidity and mortality are very low and only difficult to detect, whereas gerontology in large remains focused on the “old or older age” paradigm characterized by high degrees of dysfunction and subsequent high morbidity and mortality. Diseases in humans are generally rare before the (biological) age 40, but comorbidities are common after age 65, yet no specific field of science is focusing on how evolution resulted in a program that is extremely efficient in preventing disease for the first 40 years of life and how that program may be modulated and extended by dietary, pharmacological, or other interventions. Thus, juventology presents a complementary field to gerontology that focuses on the period of organismal life when the force of natural selection is high and body functions remain maximized.
